# Vibration reduces transcript levels of the Golgi-associated gene *GOLGA2* in HeLa cells

**DOI:** 10.17912/micropub.biology.002262

**Published:** 2026-08-11

**Authors:** Jonathan Featherston, Adriana J LaGier

**Affiliations:** 1 Biology, Grand View University, Des Moines, IA, United States

## Abstract

Mechanical vibration induces cytoskeletal remodeling, which may influence organelle-associated gene expression. Because Golgi structure depends on cytoskeletal interactions, we tested whether vibration alters mRNA levels of *GOLGA2* , a gene encoding the Golgi matrix protein GM130. Using qRT-PCR, we found that HeLa cells exposed to vibration showed reduced *GOLGA2* transcript abundance relative to no vibration controls. In contrast, transcript levels of *LMNA* , a gene associated with nuclear structure, were not significantly changed. These findings show that vibration reduces mRNA abundance of the Golgi-associated gene *GOLGA2* , indicating a selective response relative to *LMNA.*

**Figure 1. Vibration reduces mRNA transcript abundance of a Golgi-associated gene, GOLGA2 , but not a nucleus-associated gene, LMNA:** **(A)** HeLa epithelial cells were exposed to 15 minutes of ~12 mm/sec vibration (Vibration, striped bar) or no vibration (control, black bar). Relative *GOLGA2* expression was determined by qRT-PCR using the ΔΔC _t_ method, normalized to GAPDH and relative to no vibration (control). Data shown is the mean ± SEM from three independent cell cultures (N=3). * p < 0.05 no vibration vs vibration using a two-tailed *t* -test. Vibration led to a decrease in *GOLGA2* expression. **(B)** Relative *LMNA* expression was assessed from three independent cell cultures (N=3), shown as mean ± SEM. Vibration did not significantly (n.s.) change the expression of *LMNA* .

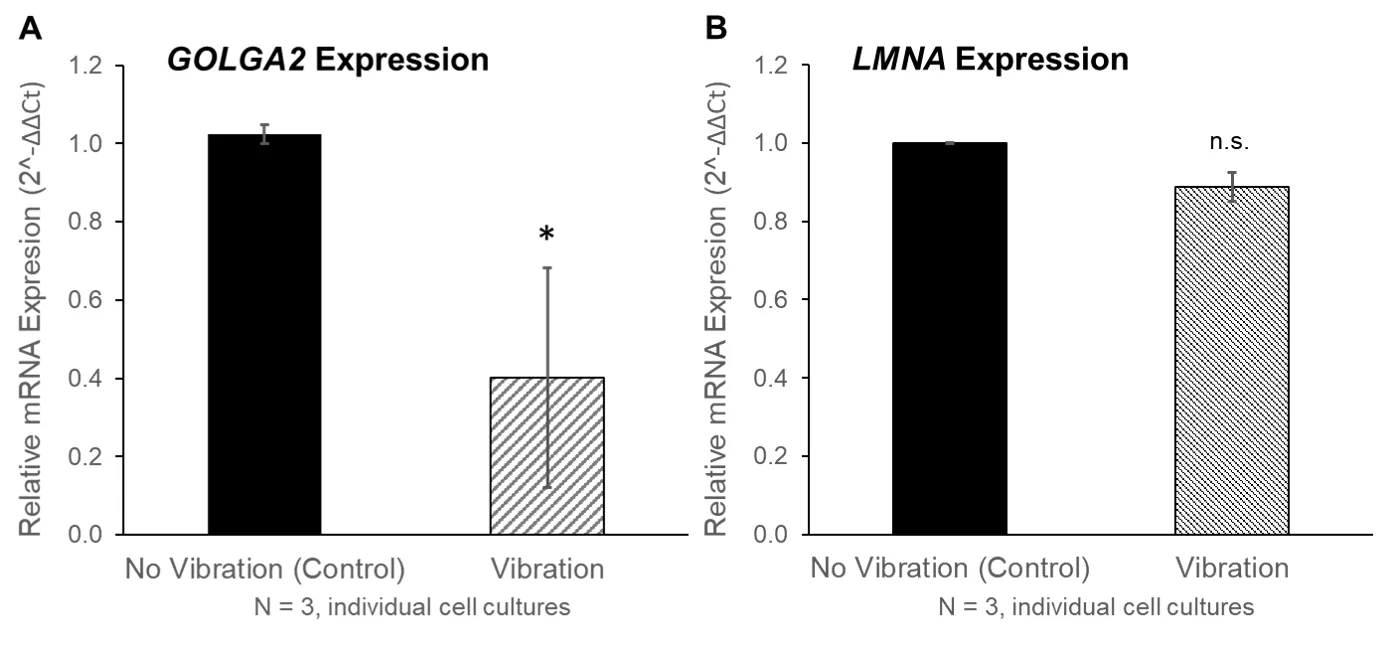



## Description

Vibration is used for medical care, including migraine treatment and neurological disorders (Juto, 2015; Ebersbach, 2008). At the cellular level, vibration has been shown to alter cytoskeletal organization and increase calpain activity, leading to actin remodeling (LaGier et al., 2022). Mechanical cues can regulate gene expression through mechanotransduction pathways (Dupont et al., 2011). Because the Golgi apparatus depends on cytoskeletal interactions for structural integrity, these changes may impact Golgi-associated proteins (Ravichandran et al., 2020; Kulkarni-Gosavi et al., 2019).

To test whether vibration affects expression of a Golgi-associated gene, we measured transcript levels of *GOLGA2* that encodes the Golgi matrix protein GM130. Also called Golgin Subfamily Member 2, GM130 assists in vesicle docking and cis-Golgi stacking.

HeLa cells were exposed to vibration (~12 mm/s, 15 minutes) or left untreated on a vibration-free table. RNA was extracted, converted to cDNA, and analyzed by qRT-PCR using *GAPDH* as a normalization control.

We observed reduced *GOLGA2* mRNA abundance in vibrated cells relative to no vibration controls. We next determined whether this effect reflected a general disruption of cellular structures by measuring expression of *LMNA* , a gene associated with nuclear structure. *LMNA* transcript levels did not significantly differ between vibrated and no vibration control cells.

These results indicate that acute (15 minute) vibration reduces expression of a Golgi-associated gene without affecting *LMNA* , consistent with the possibility that vibration preferentially affects Golgi-associated transcript levels over nuclear markers.

Mechanical agitation by vortexing generates vibration by moving fluid parallel to the cells, but it can also introduce heat, which can trigger changes in transcription levels, such as heat shock genes. Therefore, we cannot rule out that localized temperature elevations somehow contributed to altered GOLGA2 levels. To isolate mechanical forces from heat in future studies, temperature monitoring, perhaps with micro-thermocouples, should be performed.

Mechanistically, acute mechanical vibration induces rapid cytoskeletal rearrangement, including calpain-mediated actin cleavage (LaGier et al., 2022). Because Golgi structural integrity relies heavily on intact actin microfilaments and their motors, acute disruption of actin networks may trigger targeted feedback signaling to downregulate key matrix components such as GM130 ( *GOLGA2* ). Alternatively, over short timeframes, vibration could indirectly alter transcription by modulating stress-response pathways or changing the cytosolic availability and nuclear localization of mechanosensitive transcription factors (e.g., YAP/TAZ), selectively abrogating Golgi-maintenance genes while sparing nuclear envelope loci, like *LMNA* . At the genomic level, such forces may alter local chromatin accessibility, inducing facultative heterochromatin or repressor recruitment, specifically at the 9q34.11 promoter ( *GOLGA2* ) while leaving the 1q22 locus ( *LMNA* ) insulated and transcriptionally unaffected.

At the cellular level, downregulating *GOLGA2* transcripts could temporarily loosen Golgi ribbon structure or alter vesicle trafficking dynamics, serving as an adaptive stress response to safeguard the cell against vibration. At the organismal level, understanding these organelle- and locus-specific responses to mechanical force provides insight into the mechanisms underlying therapeutic vibration treatments, such as those used in neurological conditions and migraine management (Ebersbach et al., 2008; Juto & Hallin, 2015), by highlighting how physical forces selectively modulate cellular function and organelle protein quality control.

## Methods

Cell culture and vibration treatment

HeLa epithelial cells were seeded into C60 plates on three separate occasions (N=3) and grown to 90% confluency at 37°C in a humidified 5% CO _2_ incubator. At this point, HeLa epithelial cells were subjected to mechanical vibration at 12.09 ± 1.69 mm/s vibration for 15 minutes using an analog vortex mixer (V4 condition) at room temperature 20-22°C. Control cells were not vibrated (V0 condition) under identical ambient room temperature condition for 15 minutes.

RNA extraction and cDNA synthesis

Total RNA was extracted using the SurePrep™ TrueTotal™ RNA Purification Kit (Fisher Scientific) following manufacturer instructions. RNA concentration was measured using a NanoDrop Lite spectrophotometer. Samples were normalized and reverse transcribed using a High-Capacity RNA-to-cDNA kit.

qRT-PCR

qRT-PCR was performed using TaqMan® Fast Advanced Master Mix (Applied Biosystems) on a QuantStudio 5 system. Commercial TaqMan® Gene-Expression Assays (ThermoFisher Scientific) were used for *GOLGA2 (* Assay ID: *Hs01067735_g1)* , *LMNA (* Assay ID: *Hs00153462_m1)* , and *GAPDH* ( *Assay ID:* *Hs0392907_g1* ) were used. For each gene, qRT-PCR analysis was performed in technical triplicates for each of the three independent cell culture experiments. Technical triplicates were averaged before analysis. No template controls and RT(-) controls showed no amplification.

Data analysis

qRT-PCR data was normalized to *GAPDH* and expressed relative to no vibration control using the 2^-ΔΔCt method. Excel was used to run unpaired two-tailed Student’s *t* -test.

## Reagents

HeLa epithelial cells (RRID: CVCL_0030)

SurePrep™ TrueTotal™ RNA Purification Kit (Fisher Scientific)

High-Capacity RNA-to-cDNA Kit

TaqMan® Fast Advanced Master Mix (Applied Biosystems)

TaqMan probes: *GOLGA2 (* Assay ID: *Hs01067735_g1)* , *LMNA (* Assay ID: *Hs00153462_m1)* and *GAPDH* (Assay ID: *Hs0392907_g1* )

QuantStudio 5 Real-Time PCR System
